# Anti-ganglioside antibodies induced in chickens by an alum-adsorbed anti-idiotype antibody targeting NeuGcGM3

**DOI:** 10.3389/fimmu.2012.00422

**Published:** 2013-01-17

**Authors:** Marcelo D. Guthmann, Cecilia Venier, Darien Toledo, Valeria I. Segatori, Daniel F. Alonso, Leonardo Fainboim, Ana M. Vázquez, Hector Ostrowski

**Affiliations:** ^1^ELEA LaboratoriesBuenos Aires, Argentina; ^2^Laboratory of Immunogenetics, INIGEM-CONICETBuenos Aires, Argentina; ^3^Center of Molecular ImmunologyHavana, Cuba; ^4^Laboratory of Molecular Oncology, National University of QuilmesBuenos Aires, Argentina

**Keywords:** NeuGcGM3, racotumomab, anti-idiotype antibody, leghorn chickens, antibody responses, tumor antigens

## Abstract

Racotumomab is a murine anti-idiotype cancer vaccine targeting NeuGcGM3 on melanoma, breast, and lung cancer. In order to characterize the immunogenicity of alum-adsorbed racotumomab in a non-clinical setting, Leghorn chickens were immunized in dose levels ranging from 25 μg to 1600 μg. Racotumomab was administered subcutaneously in the birds' neck with three identical boosters and serum samples were collected before, during and after the immunization schedule. A strong antibody response was obtained across the evaluated dose range, confirming the immunogenicity of racotumomab even at dose levels as low as 25 μg. As previously observed when using Freund's adjuvant, alum-adsorbed racotumomab induced an idiotype-specific response in all the immunized birds and ganglioside-specific antibodies in 60–100% of the animals. In contrast to the rapid induction anti-idiotype response, detection of ganglioside-specific antibodies in responsive animals may require repeated boosting. Kinetics of anti-NeuGcGM3 antibody titers showed a slight decline 2 weeks after each booster, arguing in favor of repeated immunizations in order to maintain antibody titer. Interestingly, the intensity of the anti-NeuGcGM3 response paralleled that of anti-mucin antibodies and anti-tumor antibodies, suggesting that the *in vitro* detection of anti-ganglioside antibodies might be a surrogate for an *in vivo* activity of racotumomab. Taken together, these results suggest that Leghorn chicken immunization might become the means to test the biological activity of racotumomab intended for clinical use.

## Introduction

Racotumomab is a murine IgG1 monoclonal antibody targeting NeuGcGM3, a tumor associated antigen in human melanoma (Alfonso et al., 2002), breast cancer (Diaz et al., 2003; Guthmann et al., 2006), lung cancer (Neninger et al., 2007; Hernandez et al., 2011), as well as several pediatric tumors of such as neuroblastoma, retinoblastoma, Wilm's tumor, and Ewing's sarcoma (Scursoni et al., 2010, 2011). Racotumomab is an anti-idiotype antibody to P3, an IgM mAb raised against NeuGcGM3 (Vazquez et al., 2000). Racotumomab binds to the antigen-binding domain on P3, but detailed analysis showed that the P3 groove that interacts with the ganglioside's sialic acid is not involved in the interaction with racotumomab. Molecular modeling studies argue against a structural mimicry by the anti-idiotype antibody (Talavera et al., 2009). Rather, it is hypothesized that immunization with racotumomab induces P3-like antibodies with conserved VH germline sequences that confer them the ability to bind to NeuGcGM3, just as P3 (Boffey et al., 2005).

The immunogenic properties of racotumomab emulsified with Freund's adjuvant have been previously studied in Leghorn chickens (Hernandez et al., 2005). Results were reported for a unique 0.1 mg dose level. Racotumomab was shown to be highly immunogenic, with a marked immune dominance of racotumomab idiotype. In addition to anti-racotumomab antibodies, immunized chickens produced anti-NeuGcGM3 antibodies (Hernandez et al., 2005). As is the case in humans, NeuGcGM3 is a heterophilic antigen in chickens, which render this ganglioside a species of choice to study the immunological properties of racotumomab for clinical applications.

Racotumomab has been administered to melanoma, breast, and lung cancer patients adsorbed to aluminum hydroxide (alum). In this report we discuss the results of immunization of chickens with the same alum-adsorbed formulation used in clinical trials with cancer patients. We investigated the dose-dependence over a 25–1600 μg dose range and the booster requirements for a maximal response. We further pursued the specificity of the antibody response to a glycolylated mucin and to a NeuGcGM3-expressing tumor cell line.

## Materials and methods

### Experimental design

Six to eight week-old leghorn chickens bred under SPF conditions were immunized subcutaneously behind the neck with 25–1600 μg of 1 mg/ml alum-adsorbed racotumomab on days 0, 7, and 21. 1600 μg doses were administered by means of two 800 μl injections. 25 and 75 μg doses were administered by previously diluting the immunogen 1:10 in sterile sodium saline (immediately prior to injection) to administer 250 and 750 μl, respectively. Injections were performed with an Accura 865 microdispenser pipette (Socorex, Ecublens, Switzerland) fitted with a 25G needle.

1.0 ml blood samples were drawn at baseline and at days 14, 21, 28, and 35 from the wing vein with a 21G needle fitted to a 5 ml syringe. The blood was allowed to clot in the syringe and the serum was recovered, centrifuged and stored at −20°C till analyzed.

### Enzyme linked immune sorbent assays

Anti-racotumomab antibodies were determined by ELISA. Costar 3590 microtiter plates (Costar, Cambridge, MA) were sensitized with 10 μg/ml racotumomab or with the isotype-matched antibody iorC5 (Center of Molecular Immunology, Havana, Cuba). Serum samples were used diluted 1:10000. Bound IgG antibodies were detected with an alkaline phosphatase-conjugated goat anti-chicken IgY (Sigma Chemical Co., Saint Louis, MO) followed by 1 mg/ml p-nitrophenyl phosphate in diethanolamine buffer.

Anti-NeuGcGM3 antibodies were determined by ELISA using NeuGcGM3-coated (20 ng/well in HPLC grade methanol) PolySorp microtiter plates (Nunc, Rochester, NY). Control wells were coated with N-acetyl GM3 (Enzo Life Sciences, Miami, FL). After methanol evaporation for 2 h under vacuum, plates were blocked with 1% bovine serum albumin (cat. # A3803; Sigma Chemical Co.) in PBS. Serum samples were used either at a 1:100 dilution (for kinetics analysis) or in a two-fold dilution series starting 1:100 (for titration). Bound antibodies were detected as described above. Absorbance in control wells was subtracted to yield the specific anti-NeuGcGM3 reactivity. Titers were defined as the inverse of the dilution yielding an absorbance of 0.1 and obtained by interpolation in absorbance vs. 1/dilution plots.

Anti-mucin antibodies were determined by ELISA using Costar 3590 microtiter plates coated with bovine NeuGc rich mucin (500 μg/ml; cat. # M3895; Sigma Chemical Co.). Serum samples were used at a 1:100 dilution. Bound antibodies were detected with alkaline phosphatase-conjugated goat anti-chicken IgY (Sigma Chemical Co.) as described above.

Chickens were considered to elicit a positive antibody response if post-vaccination samples had an absorbance at least twice the value of baseline samples and superior to 0.25 arbitrary units.

### Flow cytometry

P3X63 Ag8 653 murine myeloma cells were incubated (2 × 10^5^ per tube) with 20 μl sample sera. After washing, bound antibodies were detected with an FITC-conjugated goat anti-chicken IgY (Sigma Chemical Co.) and analyzed with a FACScalibur cytometer (Becton-Dickinson, Franklin Lakes, NJ). Patients were considered responsive when two post-vaccination samples had a two-fold increase in fluorescence intensity with respect to baseline.

### Immunohistochemistry

Five-μm tumor sections from formalin-fixed, paraffin-embedded human non-small cell lung carcinoma (NSCLC) samples were processed using the usual paraffin technique. Slides were incubated for 30 min with immunized and non-immunized serum samples (1:100 in PBS) and then with biotinylated goat anti-chicken IgY (Abcam, Cambridge, MA) for 30 min, followed by peroxidase-conjugated avidin–biotin complex for 1 h. Bound antibodies were detected by incubation with diaminobenzidine substrate and tumor sections were then counterstained with haematoxylin. Pertinent specificity tests were performed, including blocking of endogenous peroxidase and use of an isotype-matched negative control antibody.

### Statistical analysis

Data from time-course experiments of antibody induction are shown as mean absorbances for each time point, where the amount of animals in each experiment is indicated in the legend to each figure. Error bars represent the standard deviation. When indicated, results are shown for representative animals, and no error bars are shown. The coefficient of variation for duplicate ELISA wells was <5%.

Maximal antibody titers are presented in scatter plots, and the amount of animals in each group is indicated in the legend. The titer median is indicated with a horizontal bar.

Anti-NeuGcGM3 and anti-mucin antibody responses in individual samples were compared in X–Y plots where the X value depicts the anti-mucin absorbance and the Y value depicts the anti-NeuGcGM3 absorbance. The Spearman test was used to assess correlation. The Spearman correlation coefficient is indicated, with the corresponding *P* value (indicating the chance that random sampling would result in a correlation coefficient as far from zero as observed).

## Results

### Kinetics of racotumomab-induced antibody response

**Figure 1:**
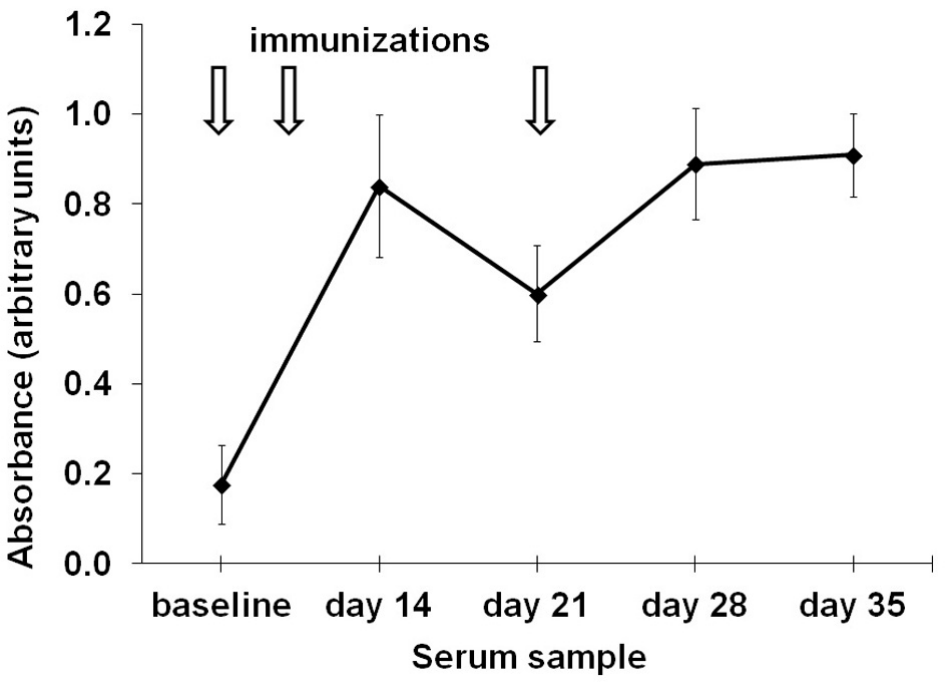
**Time course of the induction of anti-racotumomab antibodies.** Chickens (*n* = 10) were immunized with 200 μg alum-adsorbed racotumomab (arrows) and the induction of anti-racotumomab antibodies was assessed at the indicated times. The mean absorbance and standard deviation is shown.

Chicken were immunized with 200 μg of alum-adsorbed racotumomab at day 0, and boosted at days 7 and 21. Blood was drawn at baseline, days 14, 21, 28, and 35 to assess the antibody response. All chickens presented racotumomab-specific antibodies after immunization. Antibody levels peaked after two doses (day 14) and remained at the same level after an additional boost. Antibody levels decayed after the first week following each boost, and repeated boosting was required to maintain the antibody levels (Figure 1). Maximal antibody levels were most frequently observed 7 days after the previous immunization (in 65% of birds) than in time points 14 days after the immediately previous boost (the remaining 35% of birds).

**Figure 2:**
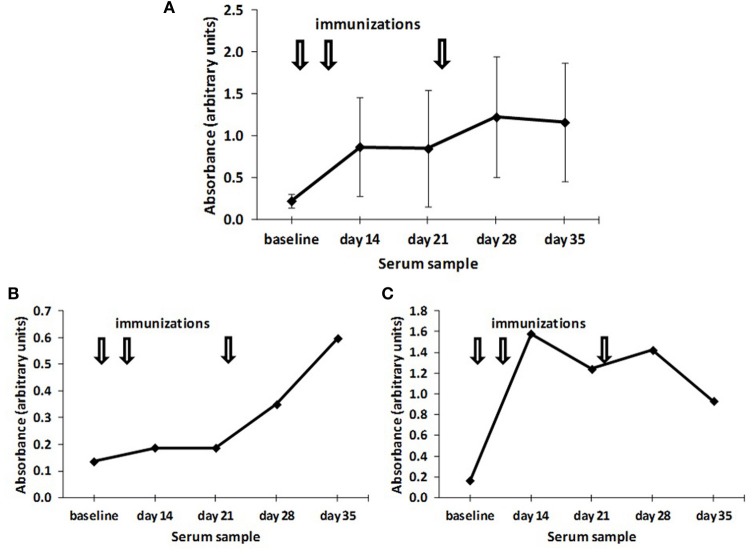
**Time course of the induction of anti-NeuGcGM3 antibodies. (A)** Chickens (*n* = 10) were immunized with 200 μg alum-adsorbed racotumomab (arrows) and the induction of anti-NeuGcGM3 antibodies was assessed at the indicated times. The mean absorbance and standard deviation is shown. **(B** and **C)** Time course of the induction of anti-NeuGcGM3 antibodies in individual chickens immunized with 200 μg alum-adsorbed racotumomab (arrows). The induction of anti-NeuGcGM3 antibodies is indicated for a representative early responder **(B)** and a representative late responder **(C)**.

Anti-ganglioside levels, in contrast, presented a very high variability in the antibody values (Figure 2A) as a consequence of a heterogeneous kinetics in antibody response. Whereas some birds showed an early response with a maximal response after two doses and a gradual reduction thereafter (Figure 2B), other birds showed an induction of significant antibody levels only 2 weeks after the last immunization (Figure 2C).

### Dose dependence of antibody response

Six cohorts of 10 chickens each received the immunization schedule described above in a dose level ranging from 25 to 1600 μg alum-adsorbed racotumomab. Serum samples were analyzed for anti-racotumomab and anti-ganglioside response. All chickens elicited anti-racotumomab antibodies in entire dose level range studied. The proportion of chickens that induced anti-ganglioside antibodies was dose dependent, with 100% responsive birds at the 200 μg dose level. The groups receiving the lowest and highest dose levels presented a lower proportion of chickens with ganglioside-specific responses (80% and 60%, respectively).

**Figure 3:**
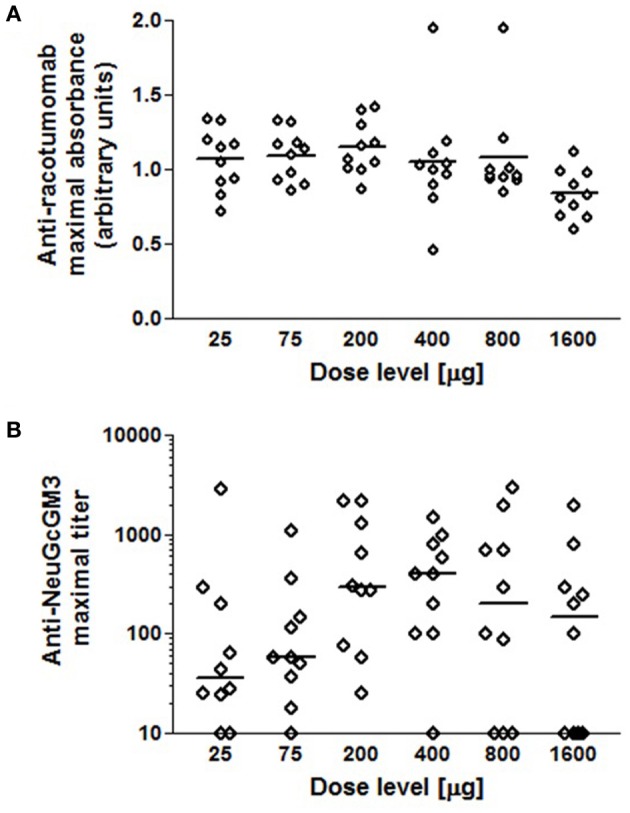
**Dose dependency of the antibody response.** Ten chickens in each dose level group were immunized as described. Sera were analyzed for anti-racotumomab and anti-NeuGcGM3 antibodies. Maximal absorbance (anti-racotumomab) or titer (anti-NeuGcGM3) were recorded for each chicken and used for comparison between dose levels. **(A)** Maximal anti-racotumomab antibodies for individual chickens receiving the indicated dose (mean absorbance is indicated with a bar). **(B)** Maximal anti-NeuGcGM3 antibody titers for individual chickens receiving the indicated dose (median titer is indicated with a bar).

The maximal antibody response for each bird was recorded for dose-response correlation. No difference was found in the anti-racotumomab response in the 25–200 μg immunogen range. Interestingly, higher dose levels (800–1600 μg) yielded a slightly weaker anti-racotumomab antibody response (Figure 3A).

The anti-ganglioside response, in contrast, showed a maximal response in the 200–400 μg dose range and a comparatively weaker response in both the lower and higher ends of the examined dose range. In the latter groups, the titer mean was lower and the fraction of birds who failed to show detectable anti-ganglioside antibodies was increased (Figure 3B).

### Immunodominance of racotumomab idiotype

**Figure 4:**
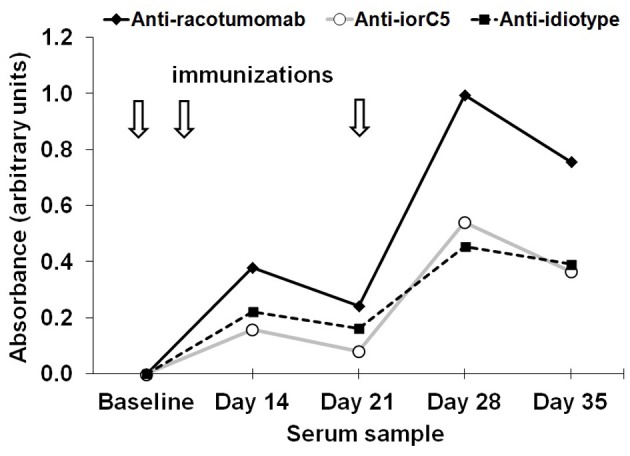
**Induction of anti-idiotype antibodies.** Time course of anti-racotumomab and anti-iorC5 (an isotype-matched control antibody) antibodies in a representative chicken in the 200 μg dose level group (*N* = 10). Immunization time line is represented by arrows. Similar immunodominance of racotumomab idiotype was observed in all other dose levels. The dashed line depicts the time course of anti-idiotype antibodies (Abs_anti-racotumomab_–Abs_anti-iorC5_).

The influence of dose-level on the immunodominance of racotumomab idiotype was assessed by comparatively determining the binding to racotumomab and to a control isotype-matched monoclonal antibody (iorC5). Reactivity against racotumomab was approximately twice than that to the isotype control at all time points, confirming that a substantial proportion of the elicited antibodies are directed toward the racotumomab idiotype and that this proportion does not vary significantly with re-immunization (Figure 4).

The anti-idiotype response (the difference of reactivity of serum antibodies to racotumomab and to isotype control) was compared throughout the 200–1600 μg dose level range and was found to closely parallel that of the anti-racotumomab response and decreased at the higher dose level (not shown).

### Specificity of anti-ganglioside response

The fine specificity of the elicited anti-carbohydrate antibodies was assessed by determining their reactivity against N-Acetyl GM3 (a widely expressed ganglioside in the surface of normal cells), against an N-glycolyl neuraminic acid-rich mucin, against a tumor cell line expressing high levels of NeuGcGM3 on its cell surface and against NeuGcGM3-positive non-small cell lung cancer (NSCLC) tissue sections.

**Figure 5:**
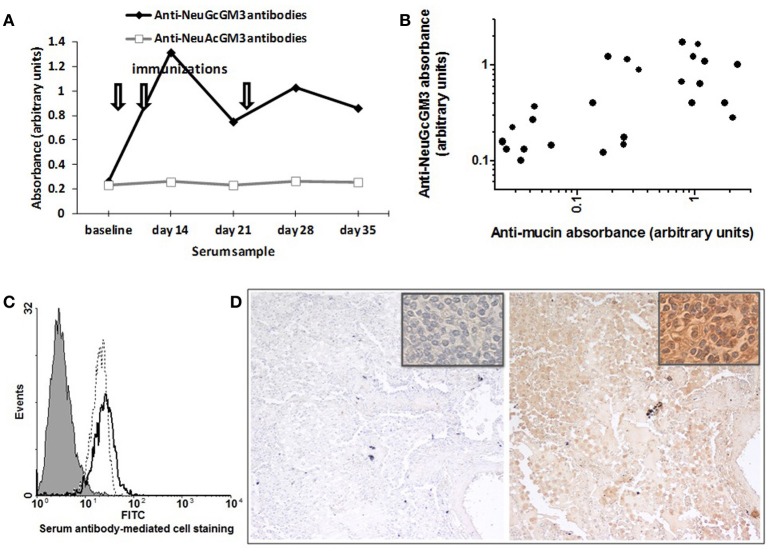
**Specificity of racotumomab-induced antibody response. (A)** Anti-ganglioside response. The time course of anti-NeuGcGM3 and anti-NeuAcGM3 antibody response is shown for a chicken immunized with 200 μg racotumomab as previously described (one chicken is shown, representative of 10). Similar results were obtained with all dose levels, with no induction of anti-NeuAcGM3 antibodies (not shown). **(B)** Anti-mucin response. Serum samples of different anti-NeuGcGM3 antibody content and from different dose level groups (*n* = 26) were tested for anti-mucin antibodies and the absorbencies for each sample was added to an x–y plot (Spearman *r* = 0.66; *p* < 0.001). **(C)** Anti-NeuGcGM3^+^ cell line antibodies. X-63 myeloma cells were incubated with serum samples and stained for cell-binding IgY antibodies. Overlaid histograms are shown for three samples of a chicken from the 200 μg dose level group. Filled histogram: baseline serum sample. Dotted line: day 14 serum sample. Solid line: day 28 serum sample. **(D)** Anti-NeuGcGM3 positive tumors response. NSCLC tumor section were incubated with baseline (left panel) and post-immunization (right panel) serum samples and stained for bound IgY antibodies. Serum of one chicken from the 400 μ g dose level group was used. Original magnification X100, X1000 (insets).

Although NeuGcGM3 and N-acetyl GM3 only differ by a single oxygen atom on the acetyl group on the amine at carbon 5 of the sialic acid, no N-acetyl GM3-specific antibodies were detected irrespective of the number of immunizations or the kinetics of anti-ganglioside response (Figure 5A).

Serum antibodies were also reactive against mucin, suggesting that sialic-acid reactivity may be maintained when the latter is present on a different molecular backbone, as is the case with Neu5Gc-rich mucin. Furthermore, the intensity of reactivity against mucin paralleled that against NeuGcGM3 (Figure 5B), arguing for a relationship between both specificities. However, whether this is the consequence of cross-reaction or epitope spreading remains to be determined.

Reactivity of serum antibodies against NeuGcGM3 was also paralleled by reactivity against the NeuGcGM3-expressing murine myeloma line X-63. Whereas serum of non-responsive chicken failed to stain the X-63 cells (not shown), sera reactive against NeuGcGM3 (as assessed by ELISA) also recognized NeuGcGM3 exposed on the cell surface (Figure 5C).

NeuGcGM3 has been described as a tumor antigen for NSCLC in humans (van Cruijsen et al., 2009; Blanco et al., 2012). Considering this, we assessed the presence, in baseline and post-vaccination serum samples, of antibodies reacting with NeuGGM3 on fixed and processed NSCLC tissue sections. As it is shown in Figure 5D, the post-vaccination serum (but not the baseline serum) was able to react with tumor sections showing a moderate to intense immunoreactivity.

## Discussion

Water in oil emulsions are very effective vaccine vehicles for improving antigen-specific humoral responses in chickens, owing to a combination of antigen residence-prolonging activity and direct immune stimulation (Jansen et al., 2007). Hernandez and co-workers have previously used Freund's adjuvant to emulsify 100 μg doses of racotumomab for immunization of Leghorn chickens (Hernandez et al., 2005). Four intramuscular injections at 2 weeks interval elicited high titer anti-racotumomab antibodies with immunodominance for its idiotype. In addition, anti-ganglioside antibodies were also induced, with specificity for NeuGcGM3 and NeuGcGM2 but not for NeuAcGM3 and NeuAcGM2, suggesting the activation of an anti-idiotypic cascade leading to a glycolylated sialic acid-specific antibody response (Hernandez et al., 2005).

However, emulsions are seldom used in the clinical setting, due to a comparatively increased toxicity. A few cancer vaccines have been combined with oil adjuvants (i.e., Montanide) or saponins (i.e., QS-21) in the need to maximize immune response in immunocompromised patients (Mesa and Fernandez, 2004). Adsorption to aluminum-based salts, on the other hand, is the most common commercial adjuvant to date, and enhances Th2 responses and antibody production (Mesa and Fernandez, 2004). Alum-adsorbed racotumomab has been extensively tested as a cancer vaccine and is presently undergoing a phase III clinical trial (ClinicalTrials.gov identifier: NCT01460472).

Because alum is less effective than microbial-derived adjuvants such as CpG ODN (de Paula et al., 2011) or Freund's adjuvant (Mayo et al., 2005; Berezin et al., 2010), we investigated the immune response to alum-adsorbed racotumomab in Leghorn chickens.

Antigen dosage is effective in poultry immunization in the 20 μg-range (Fassbinder-Orth et al., 2009). We have examined dose levels ranging from 25 to 1600 μg of alum-adsorbed racotumomab, and described a sustained anti-racotumomab response down to 25 μg of immunogen, supporting the strong immunogenicity of the racotumomab idiotype.

Maximal anti-ganglioside response was observed in the 200–400 μg range, where a responsiveness of 100% is observed and the average antibody titer reaches its peak. However, anti-NeuGcGM3 antibodies were also elicited with a dose level as low as 25 μg. The decline of the anti-ganglioside response at immunogen dose-levels in excess of 800 μg parallels that of the anti-racotumomab response, suggesting that overdosing might equally hinder both responses and that anti-racotumomab response can only be obtained at an optimal immunogen dose.

Whereas, the anti-racotumomab response is characterized most chickens by an early response followed by a sustained antibody production, the anti-ganglioside response includes both early-responders in which the titer peak after two administrations and decline thereafter, and late responders which have undetectable anti-ganglioside antibodies before the third administration. Heterogeneous kinetics in ganglioside-specific antibody response has previously been described by us in breast cancer patients receiving 16 racotumomab injections over a 50-week period (Guthmann et al., 2006). Although some patients developed anti-NeuGcGM3 IgG responses after 4 doses, late responders required up to 13 doses to elicit anti-ganglioside IgG antibodies. All patients eventually developed anti-ganglioside responses (either IgM or IgG), but the heterogeneity in the timing of the response gave ground to the acceptance of extended vaccination regimens for ensuing clinical studies. It is noteworthy that in spite of the heterogeneity in the response time frame observed in chickens, all the birds receiving the 200 μg dose responded after only three doses.

The immunodominance of the racotumomab idiotype was maintained throughout the studied dose range and the decline of the anti-idiotype response at the highest dose level (1600 μg) paralleled that of the whole anti-racotumomab response. On the other side, consistent with previous findings by Hernandez and co-workers, racotumomab immunization failed to induce anti-NeuAcGM3 antibodies (Hernandez et al., 2005) in all the studied dose range. Taken together, the results suggest that the specificity of the immune response is note affected by dose level.

The anti-NeuGcGM3 response quantitatively paralleled that against a NeuGc rich mucin, suggesting that the response elicited in chicken is most probably directed against the glycolylated sialic acid and that the chicken antibodies may bind this antigen on different molecular backbones. Most interestingly, post-vaccination sera are also able to specifically stain both the murine NeuGcGM3-expressing X-63 cell line and NeuGcGM3-positive human NSCLC tissue sections. Whether the chicken's anti-NeuGcGM3 antibodies promote oncotic necrosis of cancer cells, as described for cancer patients treated with racotumomab (Hernandez et al., 2011) remains to be established.

There is an increasing demand for the assessment of the biological activity of products with clinical application. The supervision of the manufacturing and product testing of cancer vaccines falls in the US under the scrutiny of the Center for Biologics Evaluation and Research (CBER) of the US Food and Drug Administration (FDA). Through its Guidelines for the Industry, the FDA frequently updates its policies regarding clinical evaluation (FDA, 2011). Likewise, the European Medicines Agency, through its Committee for medicinal products for human use (CHMP) has issued guidelines on adjuvants in vaccines (EMA, 2005). Although not directly targeted to cancer vaccines, these regulations are expected to be applicable to quality and non-clinical aspects of therapeutic vaccines such as anti-idiotypic vaccines (Chabicovsky and Ryle, 2006).

The results reported herein show that treatment of Leghorn chickens with alum-adsorbed racotumomab elicited a similar pattern of idiotype dominance and anti-ganglioside response as cancer patients under racotumomab immunotherapy trials (Diaz et al., 2003; Guthmann et al., 2006). Leghorn chicken immunization might therefore develop into a validated method to assess racotumomab vaccine potency, to comparatively characterize ascites- and bioreactor-derived racotumomab (Machado et al., 2011) and to certify batch-to-batch consistency in the manufacture of racotumomab intended for clinical use.

### Conflict of interest statement

M. D. Guthmann and H. Ostrowski are full time employees of Elea Laboratories, sponsor of the study described in this report. The remaining authors declare that they have no commercial or financial relationships that could be construed as a potential conflict of interest.
